# Molecular Epidemiology of Genogroup II Noroviruses Infection in Outpatients with Acute Gastroenteritis in Nanjing, China (2010–2013)

**DOI:** 10.1155/2014/620740

**Published:** 2014-07-15

**Authors:** Zhang Hong-ying, Shi Li-min, Li Wei, Wang Xuan, Qiao Meng-kai, He Min, Wang Yan, Xie Guo-xiang

**Affiliations:** Nanjing Municipal Center for Disease Control and Prevention, Nanjing 210003, China

## Abstract

*Objective.* Human noroviruses (NoVs) of genogroup II are the most common strains detected in sporadic cases of acute nonbacterial gastroenteritis in outpatients in Nanjing. To gain insight into the molecular epidemiology of GII strains, we analyzed 75 positive NoV cases from 2010 to 2013. *Methods.* The sporadic cases were detected by real-time PCR with specific primers and probes to human NoVs of genogroup I or II, human sapovirus, human rotavirus, human astrovirus, and human enteric adenovirus. Human NoVs of genogroup II were further studied by VP1 amplification (RT- PCR), cloning, sequencing, and phylogenetic analysis. *Results.* Rotavirus and human NoVs were more frequently detected in all the cases from 2010 to 2013. Human NoVs infection was more frequent since 2011 and more frequent than rotavirus infection after 2012. Out of the 75 NoV cases of genogroup II, there were 5 GII.6, 11 GII.3, and 59 GII.4. Of the 59 GII.4, 27 cases were previous GII.4.2006b strains that circulated between 2010 and 2012; while 32 cases were the newly emerging GII.4 strains GII.4.2012 from 2011 to 2013. *Conclusion.* Our data confirm other studies on the rapid emergence and displacement of highly virulent GII.4 strains.

## 1. Introduction

Nonbacterial acute gastroenteritis is commonly caused by rotavirus, human calicivirus (HuCV), human astrovirus, or enteric adenovirus [1–5]. Rotavirus is the most common cause behind acute gastroenteritis in children and the elders and HuCV is the second cause [1], while HuCV is considered the most common cause for adults [2]. In general, HuCV is considered the top cause of nonbacterial acute gastroenteritis [2–4]. HuCV consists of noroviruses and sapovirus and was mainly reported with noroviruses for their increasing importance [2–4].

Noroviruses (NoVs), members of the genus* Norovirus *of the family Caliciviridae, have a 7.5 kb to 7.7 kb single-stranded genome of positive-sense RNA which contains three open reading frames (ORFs). ORF1 encodes for the nonstructural proteins with high conservation. ORF2 codes for the major capsid protein (VP1) with the highest degree of sequence variability in the genome. ORF3 codes for the minor capsid protein (VP2) with high variability. Similar to other caliciviruses, VP1 of NoVs is most important in strain diversity. According to VP1 sequencing analysis, NoVs are classified into five distinct genogroups (genogroup I [GI] to genogroup V [GV]) with at least 32 genetic clusters [6–9]. Of these, GII.4 strains have been the predominant strains over the last decade [10]. Furthermore, the GII.4 lineage has a 1.7-fold higher rate of evolution on average within the capsid sequence and a greater number of nonsynonymous changes compared to other NoVs [11]. As a result, there should be a new GII.4 strain emergence every 2 to 3 years [10]. However, only a little has been known about the dominant circulating genotype in Nanjing, China.

To understand the epidemiologic patterns of GII sporadic cases in Nanjing, we analyzed NoV sporadic specimens collected by Nanjing Municipal Center for Disease Control and Prevention from 2010 to 2013.

## 2. Materials and Methods

### 2.1. Real-Time RT-PCR

Viral RNA was extracted in a 10% stool suspension by SuperPure System-32 automated nucleic acid extraction and purification system (Formosa Plastic Group, Taiwan) in accordance with the manufacturer's instructions. The stool suspension was 200 *µ*L and the nucleic acid elution volume was 100 *µ*L. The nucleic acid for detection of enteric adenovirus was treated with ribonuclease at 37°C for 30 mins by RNase cocktail (Ambion (Europe) Ltd., Cambridgeshire, United Kingdom). For detecting human calicivirus (HuCV), rotavirus, human astrovirus, and enteric adenovirus, we applied primers and TaqMan probes described in the literature (Table 1). For NoV GI and GII, these primers and probes targeted NoV sequences at the ORF1-ORF2 junction, a highly conserved region of the NoV genome [12]. For human sapovirus, the primers and probe targeted a conserved region of the RNA polymerase [13]. For rotavirus, primers and probes detected rotavirus nonstructural proteins 3 [14]. For human astrovirus, primers and probes focused on the 3′ end consensus region of the astrovirus genome [15]. For enteric adenovirus, the primer pair and probes were targeting conserved segments of the hexon gene of adenovirus DNA genome [16]. Amplification was carried out in a 25 *µ*L reaction volume using the Invitrogen superscript III one-step q-RT-PCR system containing 5 *µ*l of extracted sample, 0.05 *µ*M of probe, and 0.5 *µ*M of each primer. Reverse transcription was performed for 30 mins at 50°C. Platinum Taq polymerase was activated at 95°C for 2 mins, and 40 cycles of PCR were performed at 95°C for 20 s and 60°C for 30 s using an ABI 7500 FSAT SDS. Standard mode was set to collect the fluorescence of each cycle at 60°C for 30 s. The amplification results were determined as positive when Ct values were ≤37 and there was a logarithmic growth. The experiment should be repeated when Ct values were above 37 and there was a logarithmic growth curve; the amplification was determined as positive if there was still a logarithmic growth curve and negative if there was no logarithmic growth curve.

### 2.2. RT-PCR and Sequencing for NoVs of Genogroup II 

A 252-nucleotide (nt) region of the 3′ end of the VP1 gene of 75 strains was amplified with primer set using the Qiagen one-step RT-PCR kit (Qiagen Inc., Valencia, CA). The sequencing coverage was 1×. The reaction was conducted with an initial RT step at 50°C for 30 mins, followed by PCR activation at 95°C for 5 mins, then 35 cycles of amplification (20 s at 94°C, 30 s at 56°C, and 30 s at 72°C), and a final extension step for 7 mins at 72°C in a GeneAmp PCR system 9700 thermal cycler (Applied Biosystems, Foster City, CA, USA). The RT-PCR products were purified in 2% agarose gels and cloned into pGEM-T Easy vector (Promega, Wisconsin, USA). The restricted plasmids of 2011, 2012, and 2013 or PCR products of 2010 were purified by PEG precipitation and washed with 70% ethanol. The restricted plasmids or PCR products were bidirectionally sequenced on an ABI 3730XL DNA sequencer (Applied Biosystems, Foster City, CA, USA) using the BigDye terminator cycle sequencing ready reaction kit (Applied Biosystems); as a result, one direction sequencing entirely covered another direction except for samples of 2010.

### 2.3. Sequence Editing and Analysis

All sequences generated in this study were edited with BioEdit 7.0.1 [17] and analyzed with MegAlign 5.1 [18]. Phylogenetic tree was draw with the MEGA 5.0 software [19]. For the MEGA analysis, the neighbor-joining method [20] was used for phylogenetic reconstruction, with bootstrap analysis of 1000 replicates. The evolutionary distances were computed using the Kimura 2-parameter method [21]. The translated amino acid sequences were aligned by the Clustal W method algorithm in the MEGA 5.0. Phylogenetic trees were displayed with Tree-View software [22].

### 2.4. GenBank BLAST Search for Additional NoV GII.4, GII.3, and GII.6 Sequences

To compare GII.4, GII.3, and GII.6 sequences from our study with strains that have been detected globally, a GenBank BLAST search [23, 24] was conducted with parts of VP1 sequences that were generated in this study. Reference strains from NCBI website (US National Library of Medicine National Institutes of Health; http://www.ncbi.nlm.nih.gov) were selected for the phylogenetic tree; they are Hu/GII.4/Kobe034/2006/JP, Hu/GII.4/Sydney/NSW0514/2012/AU, and others as show in phylogenetic trees.

### 2.5. Nucleotide Sequence Accession Numbers

The VP1 sequences identified in this study were submitted to GenBank under the following accession numbers: KJ528320–KJ528394.

## 3. Results

### 3.1. More Frequent NoVs Infection than Rotavirus Infection in Outpatients in Nanjing after 2012 

**Figure 1:**
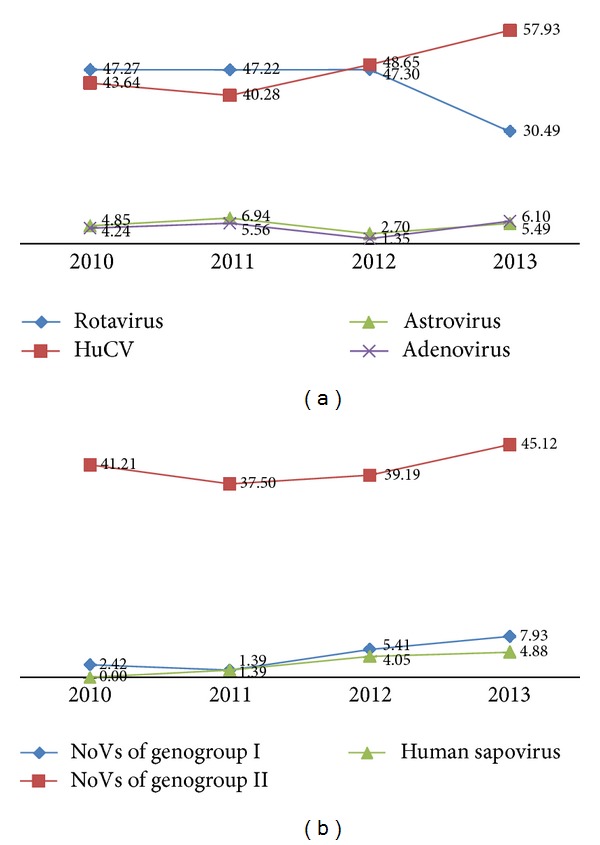
The proportion of the various viruses detected in feces of outpatients from 2010 to 2013 in Nanjing (a) and the proportion of genogroups of HuCV (b).

Between 2010 and 2013, CDC confirmed 165, 144, 148, and 164 viral positive specimens by real-time RT-PCR, respectively. In these positive strains, the majority (90%) was caused by rotavirus and HuCV each year. Before 2011, there were more cases caused by rotavirus strains than that caused by HuCV strains. During the year 2012, there was almost the same number of cases caused by these two pathogens. After 2012, more cases were caused by HuCV strains than by rotavirus strains (Figure 1(a)). For cases induced by the HuCV, the majority was caused by NoVs of genogroup II throughout these four years (Figure 1(b)).

### 3.2. Phylogenetic Relationship among GII Strains 

**Figure 2:**
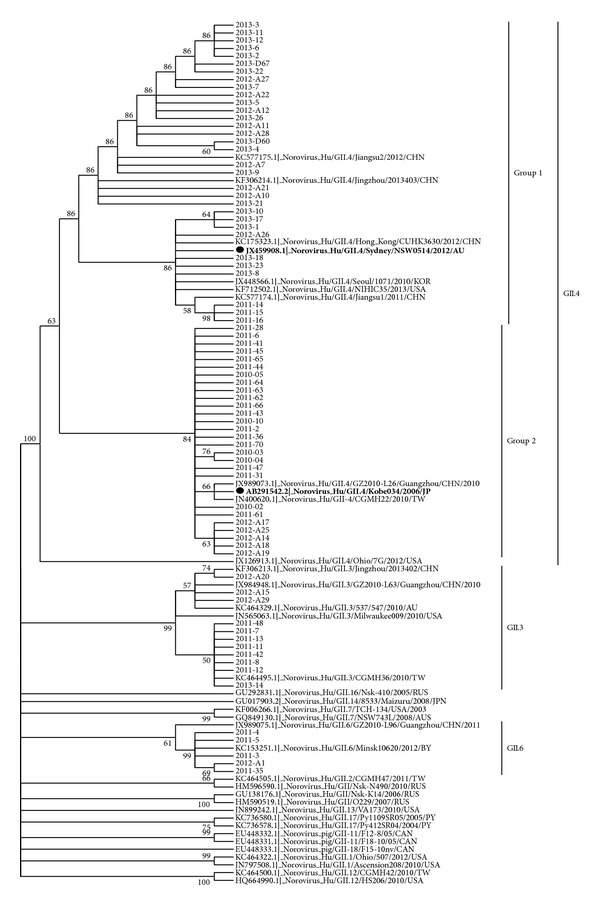
Phylogenetic analysis of identified strains based on GII (252-bp of 3′ end of VP1) of the NoV capsid gene between 2010 and 2013. The analysis involved 75 strains of Nanjing (indicated by the year of detection and strain number) and 36 additional worldwide sequences (indicated by the GeneBank accession number and strain details).

The phylogenetic analysis of 75 NoVs strains of genogroup II showed strain diversities during different years. There were 5 GII.6 and 11 GII.3 and 59 GII.4. For example, the GII.6 strains in 2011 and 2012, both were more similar to strain KC153251.1|_Norovirus_Hu/GII.6/Minsk10620/2012/BY than to strain JX989075.1|_Norovirus_Hu/GII.6/GZ2010-L96/Guangzhou/CHN/2011. Another example, the GII.3 strains of 2012 were more similar to the three strains KF306213.1|_Norovirus_Hu/GII.3/Jingzhou/2013402/CHN, JX984948.1|_Norovirus_Hu/GII.3/GZ2010-L63/Guangzhou/CHN/2010, and KC464329.1|_Norovirus_Hu/ GII.3/537/547/2010/AU than the other GII.3 strains, while the GII.3 strains of 2011 and 2013 were more similar to strain KC464495.1|_Norovirus_Hu/GII.3/CGMH36/2010/TW than the other GII.3 strains. Of the 59 GII.4 strains, there were two groups similar to strain JX459908.1|_Norovirus_Hu/GII.4/Sydney/NSW0514/2012/AU (the newly emerging GII.4 strains GII.4.2012, namely, group 1) or strain AB291542.2|_Norovirus_Hu/GII.4/Kobe034/2006/JP (the previous strains GII.4.2006b, namely, group 2), respectively. The 5 GII.4 strains in 2010 were all in group 2. The 20 GII.4 strains in 2011 were 3 in group 1 and 17 in group 2. The 14 GII.4 strains in 2012 were 9 in group 1 and 5 in group 2. The 20 GII.4 strains in 2013 were all in group 1 (Figure 2).

### 3.3. Strain Diversity of NoV GII.4 Subclusters in Nanjing

**Figure 3:**
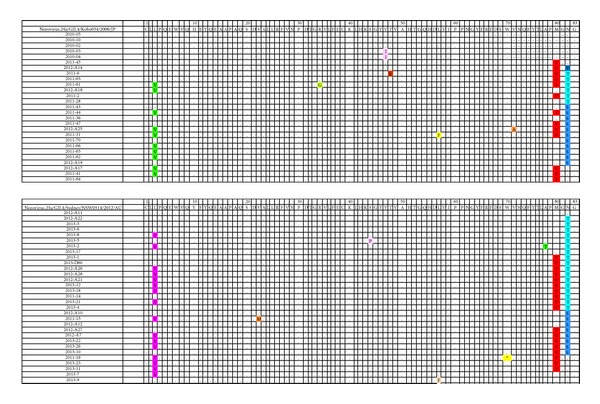
Amino acid substitutions of Nanjing strains compared to reference strains norovirus Hu/GII.4/Kobe034/2006/JP and norovirus Hu/GII.4/Sydney/NSW0514/2012/AU.

Norovirus Hu/GII.4/Kobe034/2006/JP strains were the predominant strains in 2010 and 2011. The newly variant norovirus Hu/GII.4/Sydney/NSW0514/2012/AU emerged in Nanjing in 2011 and became the predominant strain since 2012. To understand the amino acid changes of these strains, we compared the translated amino acid sequences of NoVs. Three characteristic amino acid substitutions were found in Nanjing strains not only for the former strains group of norovirus Hu/GII.4/Kobe034/2006/JP strains, but also for the newly strains group of norovirus Hu/GII.4/Sydney/NSW0514/2012/AU as shown in Figure 3. The substitutions were L453V, M530V, N532T, or N532S according the location of genome of norovirus Hu/GII.4/Kobe034/2006/JP and norovirus Hu/GII.4/Sydney/NSW0514/2012/AU.

## 4. Discussion

Our previous studies showed that the incidence rate of viral diarrhea was higher than the bacterial prevalence and increased from 2008 to 2010 in Nanjing, China [25]. In present studies, we further examined most common viral pathogens, including rotavirus, human calicivirus (HuCV), human astrovirus, and enteric adenovirus, by real-time RT-PCR, respectively, for outpatients clinically diagnosed with nonbacterial gastroenteritis from 2010 to 2013 in Nanjing. The results showed that rotavirus and HuCV were the predominant pathogens, with about ninety percent each year. The proportion of HuCV infected outpatients increased after 2012; as a result HuCV replaced rotavirus as the predominant strain in 2013. Of the HuCV, the majority was NoVs of genogroup II throughout four years. This result confirmed the importance of NoVs as genogroup II for the pathogens of nonbacterial gastroenteritis.

NoVs of genogroup II are a highly diverse subgroup of NoVs with up to 44% VP1 amino acid diversity within the genogroup [9]. And VP1 exhibits the highest degree of sequence variability in the genome [26]. Then we analyzed the sequence amplified by RT-PCR with primers targeted to VP1 epitopes of NoVs of genogroup II. The 75 strains of GII viruses showed different strain diversities and could be grouped into at least 3 distinct subclusters, namely, GII.6, GII.3, and GII.4. There were 5 GII.6, 11 GII.3, and 59 GII.4. The 5 GII.6 were closer to Minsk and Belarus strains than domestic strains. The 3 GII.3 strains of 2012 were more like both Australia strains and domestic strains, from Jingzhou and Guangzhou of China. While 9 GII.3 strains of 2011 and 2013 were closer to Taiwan strain. GII.4 was the most predominant strain throughout 2010 to 2013.

Comparison of GII.4 sequences circulating in Nanjing with sequences submitted to GenBank demonstrated that all the GII.4 strains might have a worldwide distribution. The 59 GII.4 strains were in 2 groups: 32 in the group of the newly emerging GII.4 strains GII.4.2012 (namely, group 1) from 2011 to 2013 and 27 in group of the previous strains GII.4.2006b (namely, group 2) between 2010 and 2012, respectively. A study in Shanghai showed that the predominant norovirus genotype was GII.4 and the GII.4-2006b variant was the predominant subtype both in inpatients and outpatients between 2006 and 2011 [27]. Another report in Nanjing also found that GII. 2006b is the dominant genotype in children from July 2010 to June 2011 [28]. The newly emerging strains norovirus Hu/GII.4/Sydney/NSW0514/2012/AU were first found in 2011 in Nanjing, which was much earlier than neighborhood regions [29, 30], and no other strains but the newly emerging strains were found prevalent in Nanjing in 2013, which implied the strong invasion ability of the newly emerging strains and poor human resistance to them. These results also showed evolutionary evidence for the emergence of new GII.4 subclusters (2012 sydney/AU) that gradually displaced previous GII.4 viruses in the population (2006b).

Despite the fact that the studies on NoVs for understanding antigenic sites and immunological functions are hampered by a lack of suitable animal model, some significant advances have been achieved by using virus-like particles [31] or monoclonal antibodies [32]. Besides, a number of norovirus capsid sequences have been analyzed by the evolutionary trace method and some capsid protein residues were identified that uniquely characterize different norovirus strains and form specific three-dimensional clusters that may be of functional importance in noroviruses [33]. Another comprehensive epitope analysis was conducted based on various bioinformatics technology and three conformational epitope regions of norovirus VP1 were predicted [34]. To understand the amino acid changes of GII.4 strains in Nanjing, the translated amino acid sequences were compared to the previous strain Hu/GII.4/Kobe034/2006/JP and to the new strain of norovirus Hu/GII.4/Sydney/NSW0514/2012/AU. Three characteristic amino acid substitutions were found in Nanjing strains: L453V, M530V, and N532T or N532S (according to the location of norovirus genomes Hu/GII.4/Kobe034/2006/JP and norovirus Hu/GII.4/Sydney/NSW0514/2012/AU). Whether these substitutions are involved in antigenic changes, virus fitness, or viral escape from host recognition or not still need further investigation in the future.

## Figures and Tables

**Table 1:** Primer and probe oligonucleotides used for real-time quantitative RT-PCR for NoV GI and GII, sapovirus, rotavirus, human astrovirus, and enteric adenovirus.

Primer or probe	Sequence 5′-3^′^a^^	References
Cog 1F (GI)	cgY tgg atg cgl ttY cat ga	
Cog 1R (GI)	ctt aga cgc cat cat cat tYa c	
Ring 1A (GI)	FAM^b^-aga tYg cga tcY cct gtc ca-BHQ^c^	
Ring 1B (GI)	FAM-aga tcg cgg tct cct gtc ca-BHQ	Trujillo et al. [12]
Cog 2F (GII)	caR gaR BcN atg tty agR tgg atg ag	
Cog 2R (GII)	tcg acg cca tct tca ttc aca	
Ring 2 (GII)	FAM-tgg gag ggc gat cgc aat ct-BHQ	

SapoF	gct gtt scy act ggt gca	
SapoP	FAM-cca atc saa tgt ccc tga ggc aat acg saa-BHQ	Gunson et al. [13]
SapoR	ggc atc ctg tcr ttc caa gca	

NVP3-FDeg	acc atc twc acr tra ccc tc	Freeman et al. [14]
NVP3-R	ggt cac ata acg ccc c	
NVP3-R1	ggt cac ata acg ccc cta ta	
NVP3-Probe	FAM-atg agc aca ata gtt aaa agc taa cac tgt caa-BHQ	

AV1	ccg agt agg atc gag ggt	
AV2	gct tct gat taa atc aat ttt aa	Le Cann et al. [15]
AVs	FAM-ctt ttc tgt ctc tgt tta gat tat ttt aat cac c-TAMRA^d^	

Adeno.fwd	ttc cag cat aat aac tcw ggc ttt g	Logan et al. [16]
Adeno.rev	aat ttt ttc tgw gtc agg ctt gg	
Adeno.probe1	FAM-cca tac ccc ctt att gg-TAMRA	
Adeno.probe2	FAM-cct tac ccc ctt att gg-TAMRA	

^a^R = A or G, Y = C or T, N = any, W = T, U or A.

^b^FAM, 6-carboxyfluorescein reporter dye.

^c^BHQ, black hole quencher dye.

^d^TAMRA, quencher dye.

## References

[1] Parashar UD, Hummelman EG, Bresee JS, Miller MA, Glass RI (2003). Global illness and deaths caused by rotavirus disease in children. *Emerging Infectious Diseases*.

[2] http://www.cdc.gov/norovirus/php/illness-outbreaks.html.

[3] Dolin R (2007). Noroviruses—challenges to control. *The New England Journal of Medicine*.

[4] Howley P, Knipe D, Fields B (2007). *Field’s Virology*.

[5] Chhabra P, Payne DC, Szilagyi PG (2013). Etiology of viral gastroenteritis in children <5 years of age in the United States, 2008-2009. *Journal of Infectious Diseases*.

[6] Green KY, Knipe DM, Howley PM, Griffin DE (2007). Caliciviridae: the noroviruses. *Fields Virology*.

[7] Martella V, Campolo M, Lorusso E (2007). Norovirus in captive lion cub (*Panthera leo*). *Emerging Infectious Diseases*.

[8] Wang Q, Myung GH, Cheetham S, Souza M, Funk JA, Saif LJ (2005). Porcine noroviruses related to human noroviruses. *Emerging Infectious Diseases*.

[9] Zheng D, Ando T, Fankhauser RL, Beard RS, Glass RI, Monroe SS (2006). Norovirus classification and proposed strain nomenclature. *Virology*.

[10] Zheng DP, Widdowson MA, Glass RI, Vinjé J (2010). Molecular epidemiology of genogroup II-genotype 4 noroviruses in the United States between 1994 and 2006. *Journal of Clinical Microbiology*.

[11] Bull RA, Eden JS, Rawlinson WD, White PA (2010). Rapid evolution of pandemic noroviruses of the GII.4 lineage. *PLoS Pathogens*.

[12] Trujillo AA, McCaustland KA, Zheng D-P (2006). Use of TaqMan real-time reverse transcription-PCR for rapid detection, quantification, and typing of norovirus. *Journal of Clinical Microbiology*.

[13] Gunson RN, Collins TC, Carman WF (2006). The real-time detection of sapovirus. *Journal of Clinical Virology*.

[14] Freeman MM, Kerin T, Hull J, McCaustland K, Gentsch J (2008). Enhancement of detection and quantification of rotavirus in stool using a modified real-time RT-PCR assay. *Journal of Medical Virology*.

[15] Le Cann P, Ranarijaona S, Monpoeho S, Le Guyader F, Ferré V (2004). Quantification of human astroviruses in sewage using real-time RT-PCR. *Research in Microbiology*.

[16] Logan C, O'Leary JJ, O'Sullivan N (2006). Real-time reverse transcription-PCR for detection of rotavirus and adenovirus as causative agents of acute viral gastroenteritis in children. *Journal of Clinical Microbiology*.

[17] Hall TA (1999). BioEdit: a user-friendly biological sequence alignment editor and analysis program for Windows 95/98/NT. *Nucleic Acids Symposium Series*.

[18] Burland TG (2000). DNASTAR's Lasergene sequence analysis software. *Methods in Molecular Biology*.

[19] Tamura K, Peterson D, Peterson N, Stecher G, Nei M, Kumar S (2011). MEGA5: molecular evolutionary genetics analysis using maximum likelihood, evolutionary distance, and maximum parsimony methods. *Molecular Biology and Evolution*.

[20] Saitou N, Nei M (1987). The neighbor-joining method: a new method for reconstructing phylogenetic trees. *Molecular Biology and Evolution*.

[21] Kimura M (1980). A simple method for estimating evolutionary rates of base substitutions through comparative studies of nucleotide sequences. *Journal of Molecular Evolution*.

[22] Zhai Y, Tchieu J, Saier MH (2002). A web-based Tree View (TV) program for the visualization of phylogenetic trees. *Journal of Molecular Microbiology and Biotechnology*.

[23] Zhang Z, Schwartz S, Wagner L, Miller W (2000). A greedy algorithm for aligning DNA sequences. *Journal of Computational Biology*.

[24] Morgulis A, Coulouris G, Raytselis Y, Madden TL, Agarwala R, Schäffer AA (2008). Database indexing for production MegaBLAST searches. *Bioinformatics*.

[25] Wu P, Zhang Q, Qin YP, Gao C, Su RX, Zhang HY (2011). Epidmiology of infectious diarrhea diagnosed clinically in prosperous economy aera: analysis of 1240 patients. *Chinese Journal of Nosocomiology*.

[26] Chen R, Neill JD, Noel JS (2004). Inter- and intragenus structural variations in caliciviruses and their functional implications. *Journal of Virology*.

[27] Lu L, Zhong H, Xu M (2014). Molecular epidemiology of human calicivirus infections in children with acute diarrhea in Shanghai: a retrospective comparison between inpatients and outpatients treated between 2006 and 2011. *Archives of Virology*.

[28] Li XL, Li DD, Cheng WX (2012). Molecular and epidemiological study on among children under 5 years old in Nanjing. *Zhonghua Shi Yan He Lin Chuang Bing Du Xue Za Zhi*.

[29] Shen Z, Wang G, Zai SB, Hu YW, Yuan ZH, Zhang J (2012). The emergence of novel GII.4 norovirus variant, Sydney-2012, in Shanghai, China. *Bing Du Xue Bao*.

[30] Fu JG, Ai J, Jin M (2013). Molecular characteristics of acute gastrocenteritis outbreaks caused by norovirus, in Jiangsu province. *Zhonghua Liu Xing Bing Xue Za Zhi*.

[31] Green KY, Lew JF, Jiang X, Kapikian AZ, Estes MK (1993). Comparison of the reactivities of baculovirus-expressed recombinant Norwalk virus capsid antigen with those of the native Norwalk virus antigen in serologic assays and some epidemiologic observations. *Journal of Clinical Microbiology*.

[32] Yoda T, Terano Y, Suzuki Y (2001). Characterization of Norwalk virus GI specific monoclonal antibodies generated against *Escherichia coli* expressed capsid protein and the reactivity of two broadly reactive monoclonal antibodies generated against GII capsid towards GI recombinant fragments. *BMC Microbiology*.

[33] Chakravarty S, Hutson AM, Estes MK, Prasad BVV (2005). Evolutionary trace residues in noroviruses: importance in receptor binding, antigenicity, virion assembly, and strain diversity. *Journal of Virology*.

[34] Chen L, Wu D, Ji L (2013). Bioinformatics analysis of the epitope regions for norovirus capsid protein. *BMC Bioinformatics*.

